# Marked differences between continuous long-term and clinical snapshot examinations: is the current standard of back pain diagnostics outdated?

**DOI:** 10.3389/fbioe.2024.1411958

**Published:** 2024-07-25

**Authors:** Hendrik Schmidt, Aboulfazl Shirazi-Adl, Maxim Bashkuev, Luis Alexander Becker, Matthias Pumberger, Georg N. Duda, Sandra Reitmaier

**Affiliations:** ^1^ Julius Wolff Institut, Berlin Institute of Health at Charité–Universitätsmedizin Berlin, Berlin, Germany; ^2^ Division of Applied Mechanics, Department of Mechanical Engineering, École Polytechnique, Montréal, Canada; ^3^ Center for Musculoskeletal Surgery, Charité–Universitätsmedizin Berlin, Berlin, Germany

**Keywords:** spine loading, *in vivo*, lumbar profile, kinematics, kinetics, bending moments, torsion, stiffness

## Abstract

Current clinical examination of low back pain (LBP) patients primarily relies on static clinical examinations, which rarely represent the dynamic postures patients adopt during daily activities. To gain an overview on the dynamic kinematics-kinetics changes over a day, the lumbar back kinematics of asymptomatic individuals and LBP patients were measured over 24 h, and the passively resisted bending and torsional moments were estimated. 208 asymptomatic subjects (115 females) and 116 LBP patients (71 females) were analysed. Compared to static upright standing, the mean lumbar lordosis of asymptomatic subjects drops significantly by 21° during everyday life (*p* < 0.01). Maximum bending moments of 44.0–50.6 Nm were estimated at the L2-L3. LBP patients showed significantly lower (*p* < 0.01) lumbar flattening during daily life of about 16°. Maximum bending moments of 27–52 Nm were found at the L3–L4. The initial static upright lumbar lordosis was significantly lower in LBP population (by 6°) resulting in almost similar average lumbar shapes during daily activities in both groups. The torsional movements were with 2.2° greatest in L1-L2 independent of sex (*p* = 0.19) and LBP (*p* = 0.54) with moments of 6–16 Nm. The lumbar profile and associated internal moments during daily life differ substantially from those recorded during clinical examinations. LBP patients demonstrates significantly lower lordosis at the snapshot assessment and significantly lower movement variations and internal moments during daily life. Only the dynamic long-term assessments unravelled a less flexed posture in LBP population. Apparently, such a reduced dynamic flexed posture indicates a compensatory habit for pain relief.

## 1 Introduction

The majority (70%–85%) of the global population suffers from low back pain (LBP) at some point in their life (Anderson, 1981; GBD, 2023), with 4%–25% of cases becoming chronic, depending on age and sex (Meucci et al., 2015). Both surgical and non-surgical treatments have risen disproportionately in recent years compared to treatments for other major musculoskeletal disorders affecting the hip, knee, or shoulder (Deyo and Mirza, 2006; Yoshihara and Yoneoka, 2015). Despite this rise, the success of these treatments remains moderate at best (Maher et al., 2017). Notably, 1 year after an initial episode of LBP, 67% of individuals continue to experience pain (Itz et al., 2013). This disparity indicates potential gaps in the current diagnostic and treatment approaches for LBP, which may not be sufficiently evidence-based (Deyo et al., 2009). The diversity in treatment strategies (Foster et al., 2018) and the lower rate of patient satisfaction (Itz et al., 2013) suggest limitations in our understanding and management of LBP, impacting patient outcomes.

Despite decades of research focusing on the pathology and treatment of LBP, our understanding of this disorder and the success of its treatments remain limited (Maher et al., 2017). Most research has been descriptive, and a more mechanistic understanding of the inter-relationship between spinal structures, mobility, and the etiology of pain has been relatively rare. Specifically, current diagnostics often rely on static as well as pseudo-dynamic radiological measurements taken in a single upright standing position. While these snapshot examinations are crucial for clinical observation and planning for spinal and hip surgery (Le Huec et al., 2015), they may not accurately reflect the dynamic and varied postures encountered during daily activities, potentially leading to less effective treatment strategies.

Continuous long-term monitoring of spinal kinematics could provide a superior alternative to these snapshot examinations by offering a more comprehensive overview of the biomechanical environments of the spine in both healthy individuals and those with LBP (Rohlmann et al., 2014a; Dreischarf et al., 2016a). Preliminary data indicate that the actual sagittal profile of the lumbar spine during daily activities differs significantly from data recorded during short-term clinical assessments, highlighting the potential benefits of long-term monitoring (Dreischarf et al., 2016a).

Over the last sixdecades, researchers have focused on different experimental techniques to measure and estimate spinal kinematics and kinetics *in vivo* (Dreischarf et al., 2016b). Furthermore, computer models have emerged as powerful tools in estimating spinal loads at different spine levels (Dreischarf et al., 2016b; Ghezelbash et al., 2020). Similarly to the previously described posture analyses, both *in vivo* and *in silico* studies provided only limited information on long-term exposure in everyday living. Similarly to the previously described posture analyses, both *in vivo* and *in silico* studies provided only limited information on long-term exposure in everyday living. It is noteworthy that despite their significance in physiology, mechanical loads play almost no role in current clinical diagnostic procedures.

The specific objectives of the present study were to record lumbar spine kinematics and subsequently estimate segmental rotations and passive moments over an entire 24-h period of regular daily activities in individuals with and without LBP. We aim to compare the spine kinematics and passive bending and torsional moments experienced by healthy and LBP populations in short-term versus long-term assessments. We hypothesize that:

1. Both healthy and LBP populations will experience distinct spine kinematics and bending and torsional moments in short-term versus long-term assessments.
2. There will be significant differences in long-term exposures to spine kinematics, bending, and torsional moments between healthy individuals and those with LBP.

Such comparisons will enhance our understanding of the short- and long-term alterations in LBP population, potentially paving the way to a more personalized patient stratification and more effective treatment plans.

## 2 Materials and methods

### 2.1 Study design

The working protocol of the present cross-sectional, cohort study proceeded in four steps: 1) lumbar back mobility of asymptomatic participants (Asym) as well as LBP patients was measured for short- and long-term periods. 2) The accuracy of the foregoing back and spine shape measurements was assessed in different postures by X-ray images considered as the gold standard in this step. 3) The collected kinematics data were partitioned into different lumbar spinal segments. 4) These segmental rotations were subsequently converted into segmental passive bending and torsional moments based on available reported *in vitro* and *in silico* studies. The study was prospectively registered (DRKS-ID: DRKS00027907) and performed in Germany. The Ethics Committee of the Charité–Universitätsmedizin Berlin (registry numbers: EA4/011/10, EA1/162/13) approved this study. All study participants were informed about the study’s procedure and signed a consent form.

### 2.2 Participants

Several earlier studies demonstrated that the shape of the back skin surface matches that of the underlying spine (Adams et al., 1986; Guermazi et al., 2006). We showed that such correlations are, however, rather poor in overweight and obese persons (Rohlmann et al., 2014a). To allow a sufficiently accurate estimation of the spine shape and motion by the skin surface wearable technology, only persons with a body mass index (BMI) lower than 27 kg/m^2^ were included in this study (see Table 1 for the age, body height, body weight and BMI data). The measurements were performed during regular working days from Monday to Friday. The study participants were employed mainly in the university environment (student assistants, research assistants), in the healthcare sector (doctors, nurses) and in the service sector.

**TABLE 1:** Study participants (Asym, asymptomatic; LBP, low back pain patients) mean values (standard deviation) for age, body height, weight and body mass index (BMI).

		n	Age (years)	Height (cm)	Weight (kg)	BMI (kg/m^2^)
Asym	All	208	40.3 (14.0)	173.0 (9.6)	68.0 (9.9)	22.7 (2.0)
	Females	115	40.9 (14.1)	167.4 (7.1)	62.1 (7.0)	22.1 (2.0)
	Males	93	39.5 (13.8)	179.9 (7.8)	75.4 (7.8)	23.3 (1.8)
LBP	All	116	50.2 (13.9)	171.2 (7.9)	68.6 (10.1)	23.4 (2.4)
	Females	71	49.0 (14.0)	167.4 (5.3)	64.2 (8.9)	22.9 (2.8)
	Males	45	52.2 (13.6)	177.0 (7.7)	75.7 (7.7)	24.1 (1.4)
*p-value between Asym & LBP*						
*Females*			0.280	0.912	0.804	0.420
*Males*			0.150	0.710	0.802	0.380

Asym, asymptomatic; LBP, low back pain patients; body mass index (BMI); n, number of participants; **mean values** (standard deviation).

#### 2.2.1 Asymptomatic individuals

The asymptomatic subjects did not experience pain in the lower back or pelvis in the 6 months prior to the measurements and had never underwent spinal or pelvic surgery.

#### 2.2.2 LBP patients

Patients with chronic LBP (range: 12 months to 20 years) and pain intensity (Numeric Analogue Scale (NAS) range: 5–8, where 0 represents no sensation, and 10 the worst sensation imaginable) were included. Patients with prior vertebral fractures, radiculopathies with muscular paresis or prior spinal surgery as well as non-spinal circumstances which diminishes daily activity (cardio-vascular diseases such as COPD, heart failure, myocardial ischemia, neurological disorders, malignancies) were excluded from this study.

### 2.3 Procedure

#### 2.3.1 Measurements device

**FIGURE 1:**
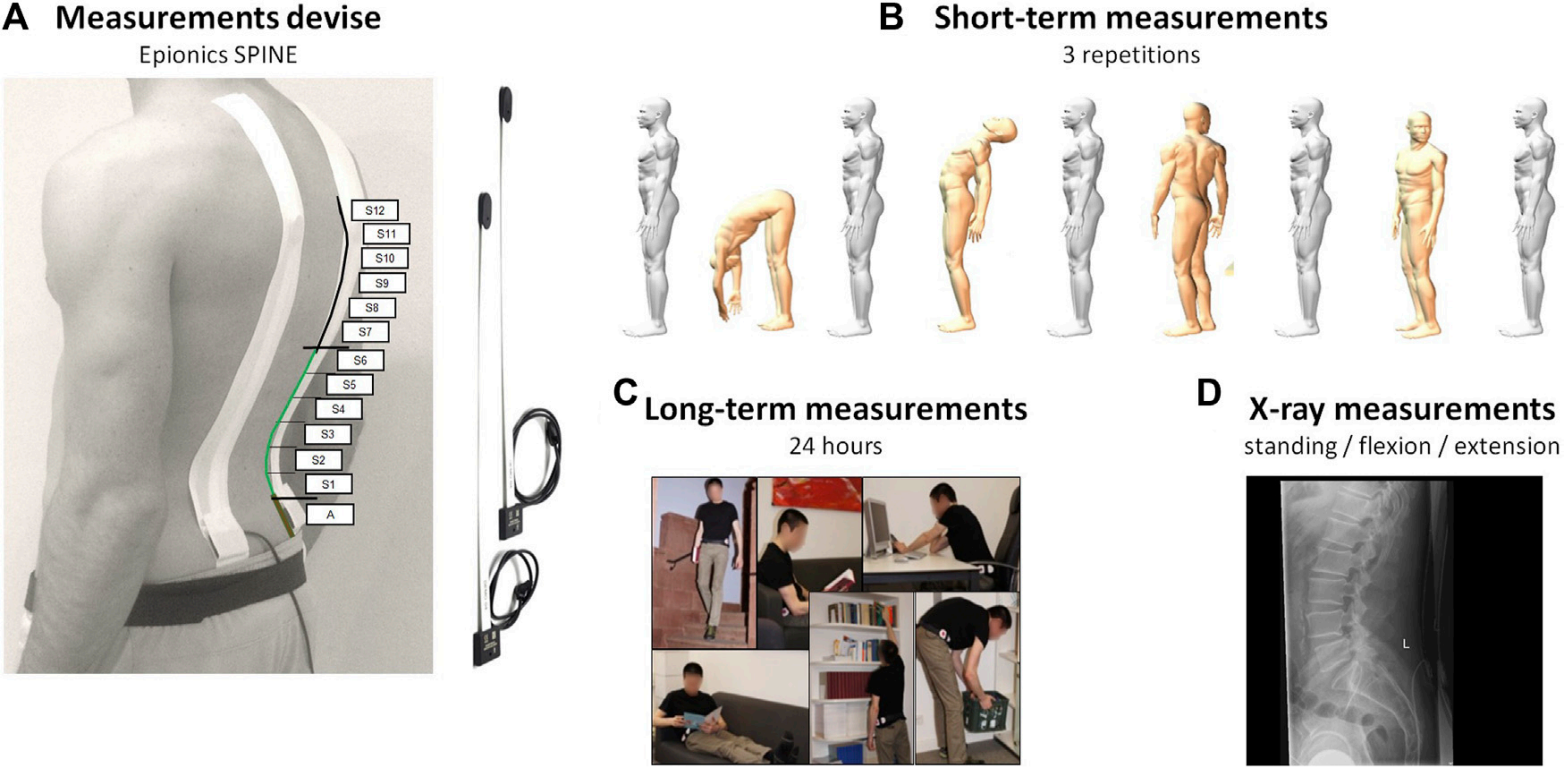
**(A)** Epionics SPINE system and the positions of the Epionics segments (S1–S12). On average, the first six Epionics segments covered the lumbar lordosis (highlighted in green) of the entire cohort. **(B)** Measurements were conducted in the upright standing as well as maximal upper body flexion, extension, left and right rotations. **(C)** Volunteers wore the device for 24 h while attending to their regular daily lives. **(D)** In total, 51 patients of the LBP group underwent radiographic measurements. Modified according to Schmidt et al. (2016).

The Epionics SPINE system (Epionics Medical GmbH, Potsdam, Germany) was used to measure, based on differential strain-gauge elements, lumbar spinal shape and rotations in the sagittal and transversal planes (Figure 1A; Supplementary Figure S1). The device consists of two flexible sensor strips, each containing twelve 2.5-cm-long segments. Our radiological examinations showed that in 92% of subjects the lower 6 Epionics’ segments correspond to the height of their lumbar spine (L1–S1) (more details are given in the section: Simultaneous X-ray & Epionics SPINE measurements). During measurements, the sensor strips were inserted into two hollow paravertebral plasters attached to the back, 7.5 cm away from the spinal column mid-line on each side. The lower end of each strip was aligned with the *posterior superior iliac spine* which is approximately in line with the first sacral vertebra. A tri-axial accelerometer was located at this end, allowing the estimation of the sacrum orientation. This acceleration sensor determined the spatial orientation of the sensor relative to the vertical direction along the earth’s gravitational field. The sensor strips were connected to a storage unit (size: 12.5 cm × 5.5 cm; mass: 80 g) that collected data at 50 Hz frequency. The sensor strips of the system exhibit high accuracy and repeatability (interclass correlation coefficient, ICC > 0.98) with test-retest reliability ICCs of >0.98 (Taylor et al., 2010; Consmuller et al., 2014). For further details, please refer to the Supplementary Material.

#### 2.3.2 Measurement protocol

Volunteers were initially instrumented with the Epionics SPINE system between 7 and 10 a.m. and were asked to perform a standardised short-term motion choreography in the sagittal and transversal planes. The choreography started with a relaxed upright standing, from which position volunteers performed maximal upper body flexion, extension, left and right axial rotations, all with knees extended and arms in the gravity direction (Figure 1B). Foregoing tasks were repeated 3 times at volunteers’ preferred speed. For guidance, the volunteers watched a video prior to the choreography that demonstrated and explained the exercises. After these short-term measurements, volunteers returned to their regular daily life activities while wearing the device for 24 h (Figure 1C).

#### 2.3.3 Data analyses

Changes in the lumbar lordosis angle of each participant was continuously calculated by considering the angles at the lower six Epionics’ segments. For segmental analyses, each Epionics segment was individually analysed to roughly estimate L1-L2, L2-L3, L3-L4, L4-L5, and L5-S1 angles. The whole lumbar lordosis and associated segmental angles served as reference values for subsequent analyses.

The motion data from each participant were collected over 24 h, and the temporal variations in lordosis and segmental rotational angles were individually calculated at each instance of time. Pure flexion and extension of the upper body was characterized by a symmetrical motion in the sagittal plane with almost identical readings at the left and right sensors (segmental differences ≤ 0,2°). Here, the lordosis angle at each time frame was determined as the sum of the angles at associated sensor segments. Asymmetric motions of the upper body led to different lordosis angles on the left and right sides of the back. These differences were taken as an Euler angle approximating the axial rotation. Finally, the duration of time spent within various intervals of segmental angles were calculated.

#### 2.3.4 Simultaneous X-ray & epionics SPINE measurements

To evaluate the validity of the Epionics SPINE system in assessing lumbar spine shape and angles in the upright standing, and flexion postures, a subgroup of LBP patients underwent both back shape and radiographic measurements simultaneously (Figure 1D). First, a standard erect standing lateral radiograph of the lumbar spine was taken of each individual. For this, each patient was instructed to stand on both feet with straight knees and hands on top of head to avoid obscuring the spine. Subsequently, each patient performed a forward flexion to the limit or as soon as a discomfort was felt.

Linear regression analysis (Pearson correlation coefficient) and the root-mean-square-error (RMSE) was used to determine the strength of linear relationship and the average deviation, respectively, between the radiographic and Epionics SPINE lumbar lordosis in the upright standing and flexion posture. The criterion for a statistical significance was set at *p* ≤ 0.05.

#### 2.3.5 Estimation of segmental sagittal and transverse moments

**FIGURE 2:**
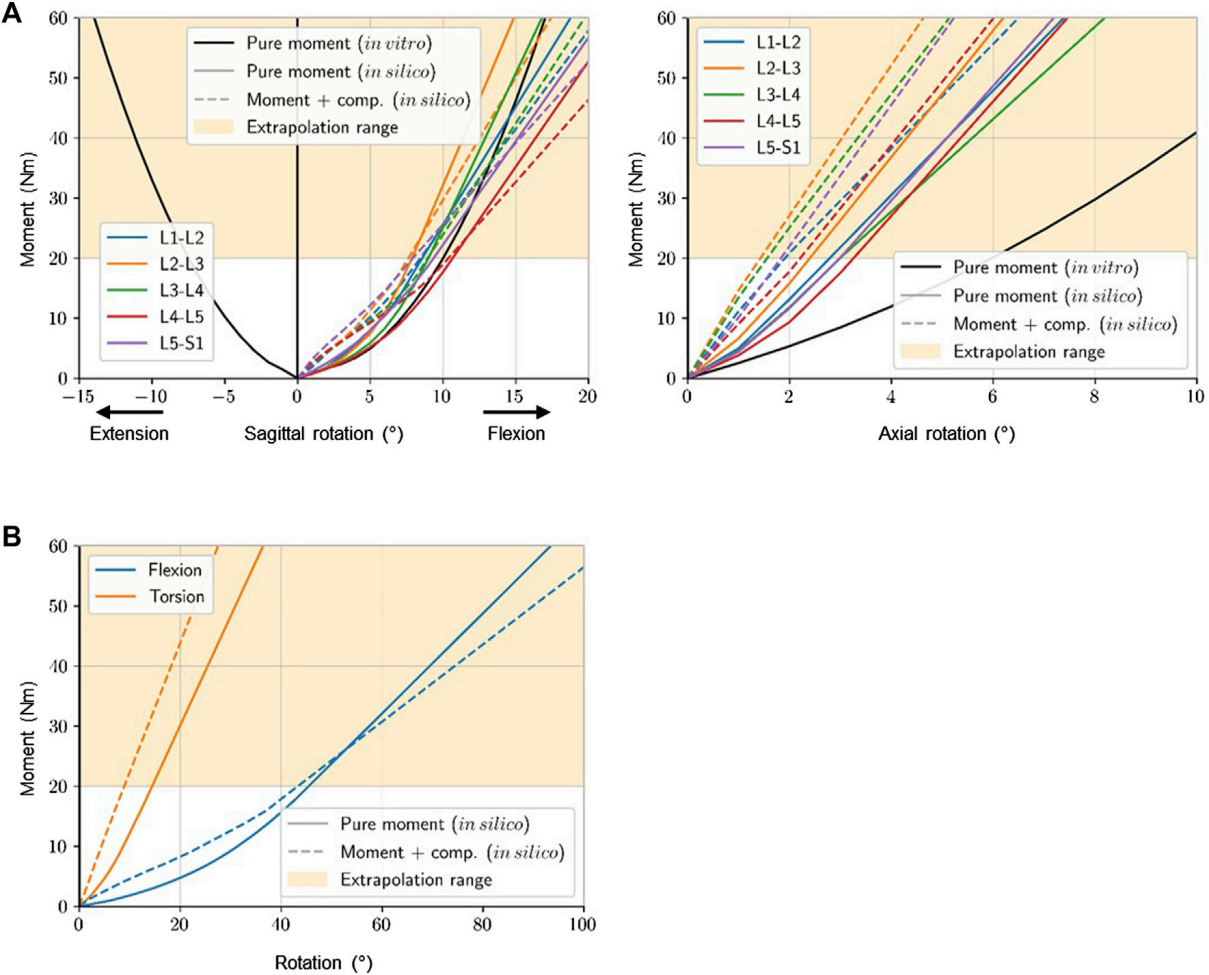
Different segmental **(A)** and lumbar **(B)** moment-rotation curves observed *in vitro* and *in silico*. Data taken from: Heuer et al. (2007) – *in vitro* & Shirazi-Adl (2006) - *in silico*. Stiffness curves were extrapolated to the maximum angle measured in daily life.

To estimate the passive segmental sagittal and transverse moments, we considered reported static moment-rotation curves determined *in vitro* (Heuer et al., 2007) (hereon referred to as “pure moment (*in vitro*)”) and *in silico* (Shirazi-Adl, 2006) (hereon referred to as “pure moment (*in silico*)” and “moment + comp. (*in silico*)”) to estimate resistant moments (Figures 2A, B). The *in vitro* study examined L4-L5 segmental stiffness in flexion, extension, and axial rotation without axial compression (*pure moment*) of specimens at a median age of 52 years (range: 38–59 years). The *in silico* study investigated the role of compression loads (0 N: “pure moment”, 2700 N: “moment + comp.”) on the lumbar and segmental stiffness in flexion and axial rotations.

The bending and torsional moments were subsequently calculated using the segmental kinematic data recorded with the Epionics system over 24 h along with foregoing measured and predicted moment-rotation curves.

### 2.4 Statistical analysis

Descriptive statistics (mean and standard deviation) were analysed, and normal distribution was tested for each age, sex, pain, and segment group by using the Kolmogorov–Smirnov test. Furthermore, Levene’s test was used to examine the equality of variances. The average lumbar lordosis and the flexion rotation from short-term measurements were specifically compared to the corresponding values from the long-term measurements using paired *t*-tests. Segmental differences between asymptomatic subjects and LBP patients as well as between the sexes in segmental moments and posture were evaluated using unpaired *t*-test. Correlations were performed using Pearson’s correlation coefficient. A *p*-value of <0.05 was considered statistically significant. Data were analysed using SPSS 27.0 (IBM SPSS Statistics for Windows NY, United States).

### 2.5 Role of the funding source

The funder of the study did not participated in the study design, data collection, data analysis, data interpretation, and review and approval of the manuscript.

## 3 Results

Between September 15, 22, and March 23, 208 asymptomatic subjects (115 females) and 116 LBP patients (71 females) were included (Table 1).

### 3.1 Back surface vs. spine X-ray shape measurements

**FIGURE 3:**
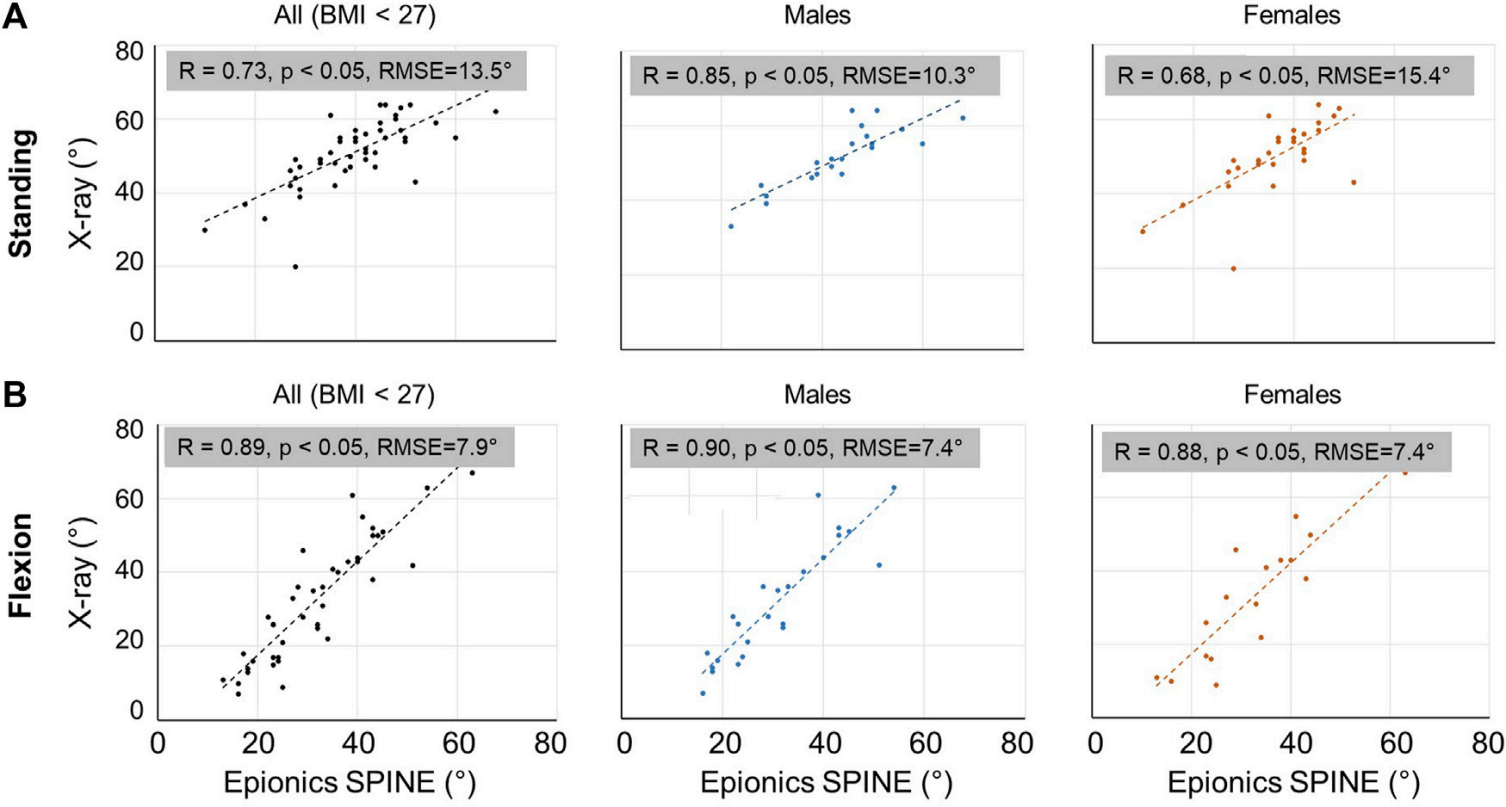
Scatter plot on the correlations between X-ray spine and back shape measurements for the entire sample size as well as for males and females separately in the upright standing **(A)** and flexion **(B)** postures. Lumbar Range of Motion (RoM) is the total rotation from standing to flexion of L1-S1.

First, we compared the classical clinical upright standing X-ray against surface measurements with the wearable device in 51 LBP patients (22 females). In the upright standing, there was a strong, significant correlation (R = 0.73) between the Epionics SPINE and the X-ray recorded lumbar lordosis in all subjects (Figure 3A). The correlation was higher in males (R = 0.85) than in females (R = 0.68). RMSE of the total lumbar spine reached 13.5° in all subjects, 10.3° in males and 15.4° in females. Flexion movements resulted in even greater correlations compared to the upright standing; in the entire population (R = 0.89, RMSE = 7.9°), in males (R = 0.90, RMSE = 7.4°) and in females (R = 0.88, RMSE = 7.4°) (Figure 3B).

### 3.2 Diurnal sagittal posture and resulting passive bending moments

**FIGURE 4:**
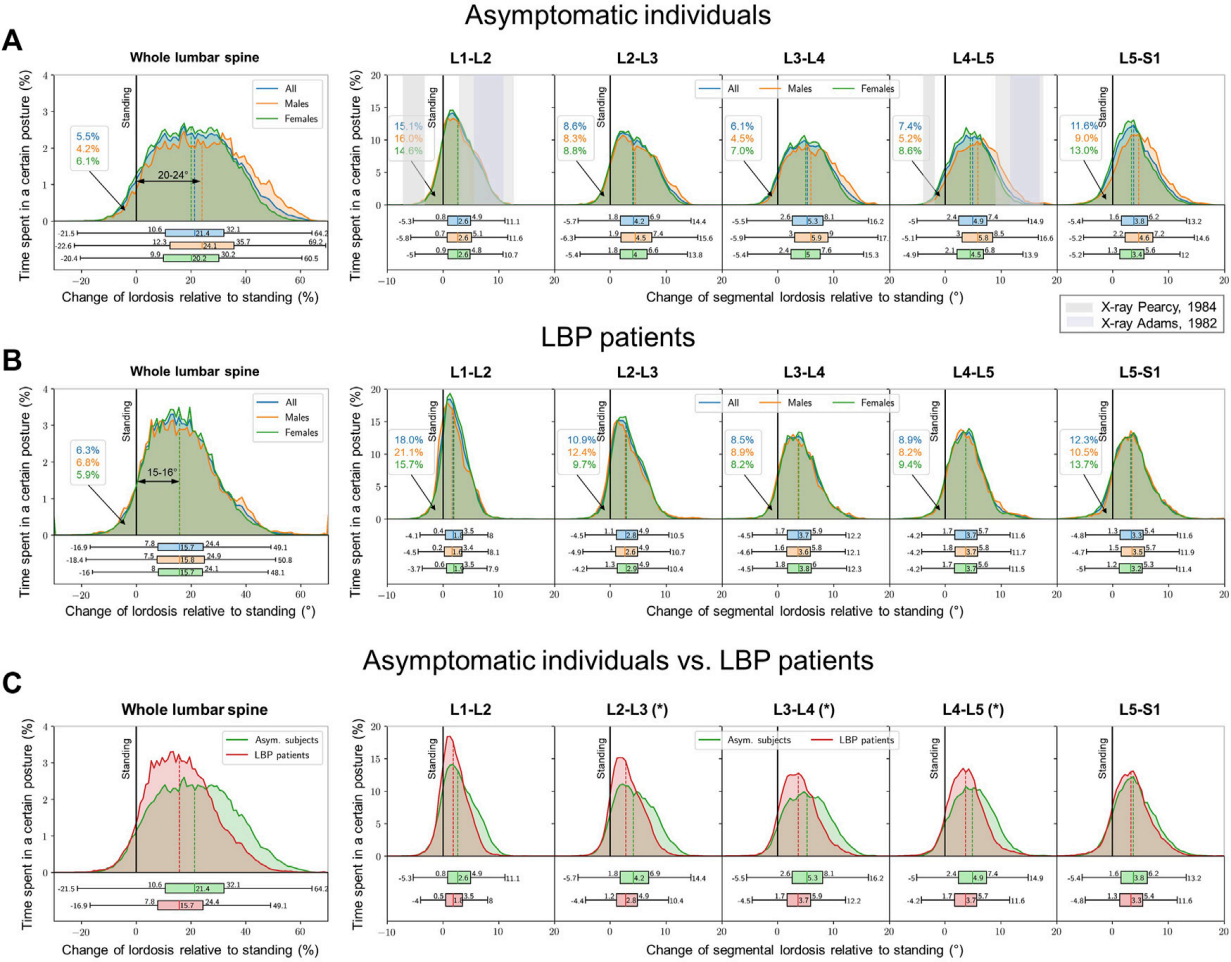
Lumbar lordosis distribution within 24 h of measurements (left). A change in the lumbar lordosis of 0° highlights the reference conventional snapshot value in the upright standing. Values > 0° indicate flexed postures with reduced lordosis whereas values < 0° indicate more lordotic postures. Segmental distributions for both cohorts (right figures). **(A)** asymptomatic subjects, **(B)** LBP patients, **(C)** LBP patients in comparison to the asymptomatic control [graphs of (**A, B)** are illustrated both on top of each other]. The snapshot lordosis angle of LBP patients in the upright standing was approximately 6° smaller than that of the asymptomatic cohort. Grey zones in **(A)** illustrate the segmental mobility in maximum voluntary flexion-extension as reported by X-ray studies of Pearcy and Tibrewal (1984) (lighter grey) as well as Adams et al. (1986) (darker grey). * indicates significant differences (*p* < 0.05).

In the snapshot upright standing measurements, asymptomatic males (32.5° ± 10.2°) and females (33.8° ± 8.4°) showed no significant differences in the lumbar lordosis (*p* = 0.195). Over the 24 h period, however, median lordosis significantly (*p* < 0.001) dropped to 26.1° ± 8.4° in males and 22.2° ± 6.8° in females (Figure 4A). Females (9.3° ± 5.1°) exhibited a slightly, but significantly larger drop in median lordosis during diurnal measurements than males (6.4° ± 6.5°) (*p* = 0.002). During the entire 24 h period, asymptomatic males and females experienced greater lumbar lordosis (compared to the upright standing) only for about 4% and 6%, respectively, of the total duration.

In contrast to the asymptomatic group, the snapshot standing lordosis in LBP males (20.2° ± 11.9°) was significantly lower (*p* = 0.041) from that in LBP females (23.7° ± 13.6°). The lordosis angle in the entire cohort of patients with LBP in upright standing was significantly (*p* < 0.01) smaller by about 6° compared to the asymptomatic population (Figures 4B, C). The LBP patients (−16.9°–49.1°) also displayed a narrower range of the lumbar lordosis during the entire day in contrast to the asymptomatic population (−21.5°–64.2°) (Figure 4C). The diurnal median lumbar lordosis compared to asymptomatic subjects (15.7° vs. 21.4°) was also significantly smaller (*p* = 0.038). Like asymptomatic subjects, the LBP subjects spent only about 6% of the whole 24 h period in more lordotic postures relative to that measured in the upright standing posture.

The decrease in the lumbar lordosis in asymptomatic subjects occurred predominantly in the middle lumbar levels, was not sex-dependent and caused an average segmental flexion angle of 4°–5° (Figure 4A). Maximum segmental flexion angle of 17° occurred in the middle and lower lumbar segments. LBP patients with 1.8°–3.7° showed significantly lower segmental flexion angles in L2–L3, L3–L4 and L4–L5 compared to asymptomatic subjects (*p* < 0.05). Here, too, no sex dependency was observed (Figures 4B, C).

**FIGURE 5:**
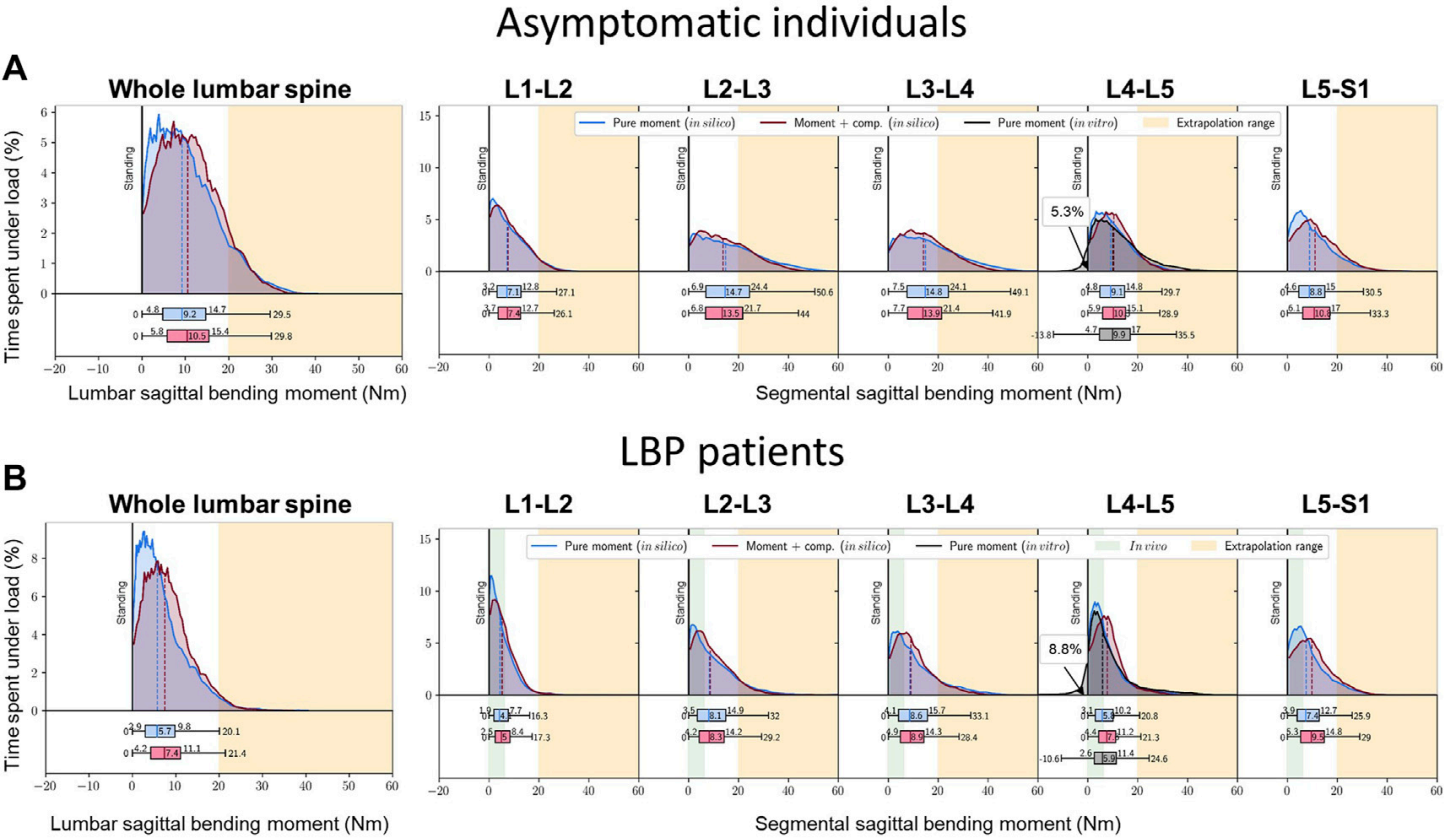
Percent time over 24 h undergoing varying flexion moments in **(A)** asymptomatic subjects, **(B)** LBP patients. The residual (initial) moments at the upright standing are taken as reference baseline here. Due to missing stiffness data, the extension moments in more lordotic postures are only given for the L4-L5 segment. Green marked area: *in vivo* measurements via instrumented vertebral body replacements (Rohlmann et al., 2014b).

The entire lumbar spine was subjected to median flexion moments of 9.2–10.5 Nm (max: 29.5–29.8 Nm). During diurnal activities, the lumbar ligamentous segments resisted median flexion moments of 7.1–7.4 Nm at L1-L2, 13.5–14.7 Nm at L2-L3, 13.9–14.8 Nm at L3-L4, 9.1–10.3 Nm at L4-L5 and 8.8–10.8 Nm at L5-S1. Maximum flexion moments of 44.0–50.6 Nm were estimated at the L2-L3 segment (Figure 5A). Because the diurnal segmental postures were not sex-dependent and identical stiffness curves were applied for both sexes, the estimated moments were not further subdivided by sex.

With smaller median flexion angles, LBP patients underwent significantly lower median passive lumbar flexion moments of 5.7–7.4 Nm (max: 20.1–21.4 Nm) and segmental flexion moments of 4.1–5.0 Nm at L1-L2, 8.1–8.3 Nm at L2-L3, 8.6–8.9 Nm at L3-L4, 5.8–7.6 Nm at L4-L5 and 7.4–9.5 Nm at L5–S1 (*p* = 0.028) (Figure 5B). Again, the middle segments were most affected with peak bending moments of 28.4–33.1 Nm at L3-L4. The foregoing estimated bending moments exceeded, for the most part, the range of loads previously measured *in vivo* via instrumented vertebral body replacements (green marked area) (Rohlmann et al., 2014b).

### 3.3 Diurnal transverse rotations and estimated passive torsional moments

**FIGURE 6:**
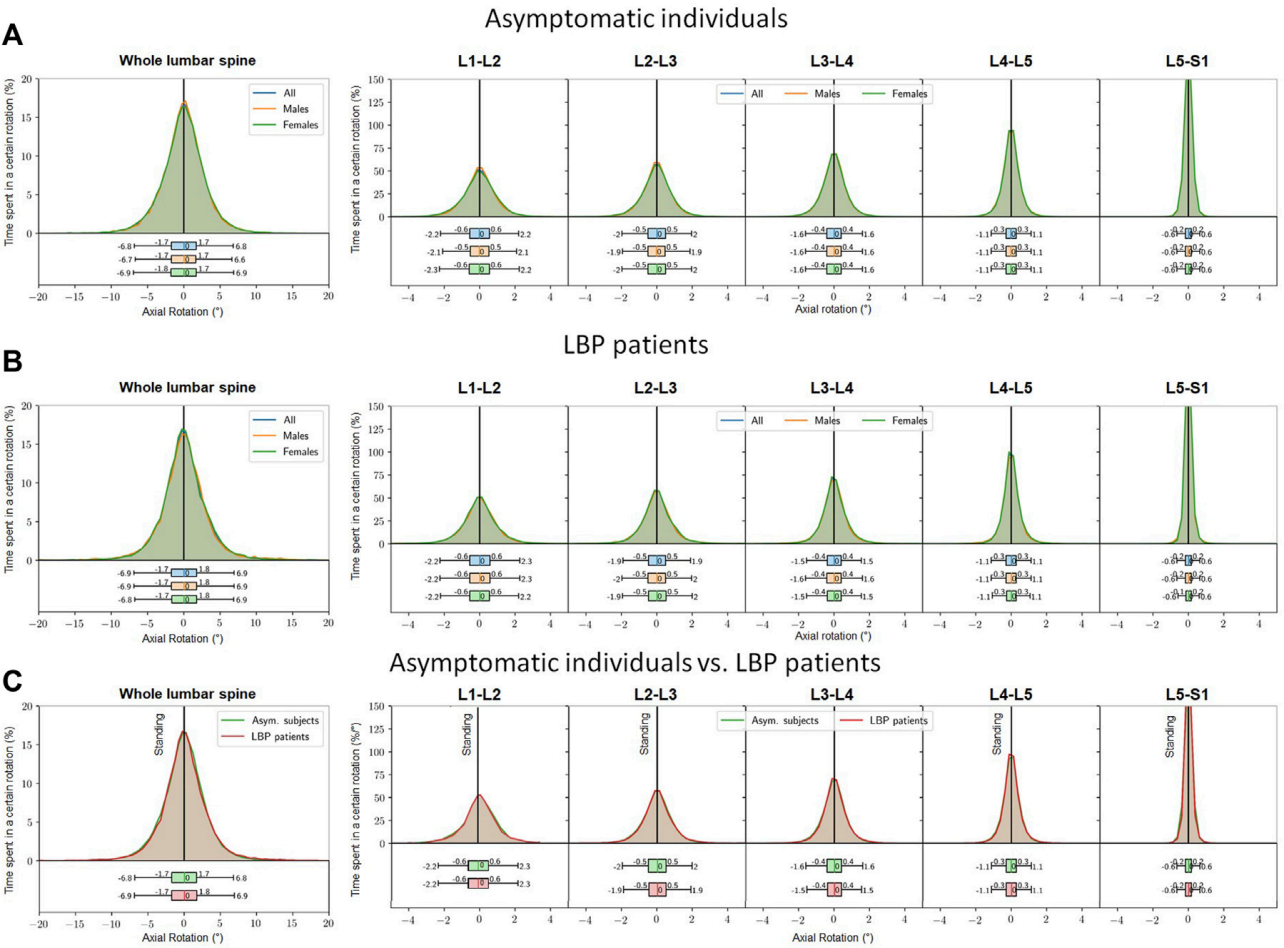
Percent time spent at a lumbar axial rotation within 24 h (left). The reference value of 0° corresponds to that at the snapshot upright standing posture. Values >0° indicate axial rotation to the right side, <0° indicate axial rotation to the left side. Segmental distributions (right figures). **(A)** asymptomatic subjects, **(B)** LBP patients, **(C)** LBP patients in comparison to the asymptomatic control (graphs of a and b are placed on top of each other).

During the entire day, both asymptomatic and LBP patients experienced similar lumbar right/left rotations with peak of approximately 10° (Figure 6A). Angles were greatest at the upper segments, up to 2.3°, and least in the lowest segment, with a maximum of 0.6° independent of sex and pain. In both asymptomatic subjects and LBP patients, there was a symmetry between the rotation to the right and left sides (Figures 6B, C).

**FIGURE 7:**
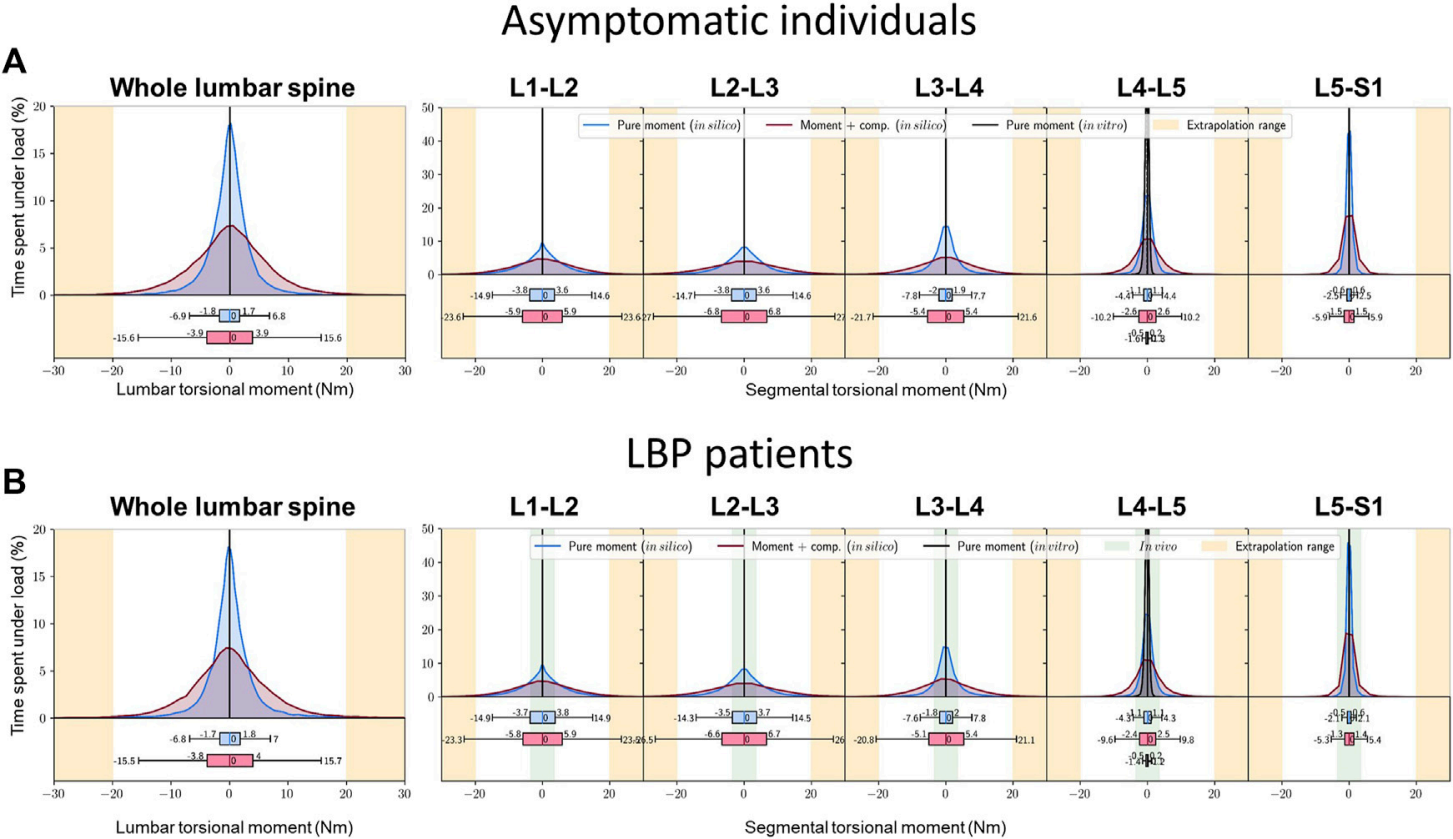
Percent time experiencing various torsional moments at the whole lumbar (left) and segmental levels (right figures), depending on the torque-angle model used over 24 h **(A)** asymptomatic individuals, **(B)** LBP patients. Green marked area: *in vivo* measurements via instrumented vertebral body replacements (Rohlmann et al., 2014b).

The lumbar spine was subjected to axial torques of 6.8–15.6 Nm. The peak moment in spinal segments occurred at L2-L3 with 14.3–26.0 Nm followed by L1-L2 with 14.9–23.3 Nm, L3-L4 with 7.6–21.1 Nm, L4-L5 with 1.2–9.8 Nm, and finally L5-S1 with 2.1–5.4 Nm, which remained the same in both healthy and LBP groups (Figures 7A, B). These estimated torsional moments fell within the range of loads previously measured *in vivo* via instrumented vertebral body replacements (green marked area) (Rohlmann et al., 2014b).

## 4 Discussion

Although effective prevention and treatment of LBP are of utmost priority, to date little is known about the time profile of lumbar spinal posture and movement in daily activities in both asymptomatic individuals and LB patients. Even less is understood about the spinal loads experienced by these groups throughout their daily activities. Consequently, the present study aimed to record kinematics and estimate internal passive moments of the lumbar spine in cohorts of healthy individuals and those with LBP during regular activities of daily living. Our hypotheses were partially confirmed, as significant differences were observed between short-term clinical assessments and long-term daily life outcomes in both healthy and LBP populations. Specifically, differences were noted in (1) kinematics and (2) spinal bending in the sagittal plane, but not in the transverse plane. Additionally, significant differences were identified between the two groups in terms of sagittal angles and moments, both statically and over time.

Sex differences in lumbar posture and kinematics have been extensively investigated, with many studies indicating no significant differences in lumbar lordosis between sexes during standing (Dvorak et al., 1995; Gelb et al., 1995; Boulay et al., 2006). However, our findings question the validity of extrapolating these results from the snapshot assessments to the daily life and long-term measurements. Specifically, long-term measurements revealed that females exhibited slightly but significantly greater lordosis than males, contrasting with previous short-term static assessments. Consequently, a valid and meaningful evaluation of crucial postural parameters as well as of the individual spinal function necessitates long-term analyses of data collected during daily life activities.

Our results show substantial differences in the daily time profile of the sagittal posture in between asymptomatic subjects and LBP patients. To explain the relationship between pain and motion adaptation, it has been proposed that the presence or fear of pain increases muscle co-activity, which enhances joint stiffness and may cause ischemia, leading to the stimulation of nociceptive afferents (Graven-Nielsen and Mense, 2001). Other theories emphasize the role of pain avoidance and cognitive-emotional mechanisms in reducing movement and function (Vlaeyen and Linton, 2000). The underlying pathophysiology is undoubtedly complex, stemming from a combination of these mechanisms and varying among individuals, potentially influenced by social and cultural factors. Recent comprehensive theories suggest that while movement restrictions and perturbations are adaptive measures to protect damaged joints and reduce pain, they can, over time, lead to undesired functional consequences and delay repair (Hodges and Smeets, 2015). Interestingly, experimental induction of LBP typically results in more protective measures, though the patterns of muscle activity modification vary between individuals (Hodges et al., 2013). Therefore, movement adaptation, initially a protective attempt to prevent further damage or pain, may eventually become part of the problem, causing undesirable alterations and further deterioration.

Interestingly, our results showed no significant differences in the transverse rotation during daily activities. This is likely due both LBP patients and healthy subjects performing rather little rotational movements in everyday life, thus not reaching pain-triggering thresholds.

The kinematic long-term measurements have some limitations. Firstly, it should be noted that the Epionics SPINE system measures the profile of the back rather than directly assessing the curvature of the lumbar spine itself and the orientation of the sacrum. However, we observed a significant correlation between lumbar lordosis estimated via back shape and radiologically determined lordosis, specifically among LBP patients with a BMI <27.0 kg/m^2^ (Figure 3), consistent with previous studies (Adams et al., 1986; Stokes et al., 1987; Guermazi et al., 2006). Notably, in overweight and obese individuals, this correlation diminishes, prompting our inclusion criteria to ensure robust alignment between back shape and spinal structures, restricting enrollment to subjects with BMI <27.0 kg/m^2^ across both cohorts. The sensor stripes are seated inside hollow plasters taped along the subject’s back, allowing the sensors to move relative to the skin surface. Longitudinally aligned elastic fibers within the plasters enable stretching and shortening during flexion and extension maneuvers. The plasters can elongate by up to 50% (24 cm). To prevent distortion of the sensor strips perpendicular to the back, the plasters exhibit minimal compliance in the transverse direction, ensuring close adherence of the sensors to the subject’s skin. External pressure applied to the sensor strips, such as from a chair backrest, has the potential to cause movement of the back that can be detected by the sensors. Furthermore, despite potential movement of the sensor strips within the plasters and their stiffness relative to the skin, the measurement’s sensitivity to skin movements is minimized. The Epionics Spine system validation included standing and flexed postures based on radiological images but did not encompass extension motions, as functional X-rays in extension were not part of routine clinical diagnostics. Given that subjects engage in extension movements for only about 5% of daily activities, this limitation was deemed minor.

The study captured data over a 24-h period, which is a long time-frame for clinical diagnostics. However, even this does not allow conclusions to be drawn about changes over longer durations or correlations with long-term patient outcomes. This restricts the comprehensive understanding of the clinical relevance of the biomechanical differences observed. Future research should address these aspects to elucidate the full spectrum of implications for long-term patient management and outcomes in the context of LBP.

The estimated bending moments in this study often exceeded the range of moments measured *in vivo* via instrumented vertebral body replacements (indicated by the green-marked area in Figures 5, 7) (Rohlmann et al., 2014b). These patients were treated with a vertebral body replacement combined with a posterior fusion. Consequently, the reported measurements do not represent moments resisted by the entire instrumented passive spine and are likely influenced by patient anxiety and kinematic adaptations due to the surgical treatment and the observational conditions under which movements were performed.

The peak bending moments on the lumbar spine in asymptomatic individuals and LBP patients reached 44.0–50.6 Nm and 28.4–33.1 Nm, respectively at L3-L4 during everyday activities (Figure 5). Earlier *in vitro* studies often considered much smaller moments, well short of the segmental elastic limit (Markolf, 1972; Lin et al., 1978). The only comparable data are those of Miller et al. that reported ‘no overt signs of failure’ in lumbar segments at 70 Nm of bending, and flexion angles between 11.7° and 13.8° (Miller et al., 1986).

Our results indicate that internal bending moments in daily life are, on average, smaller in LBP patients compared to asymptomatic individuals. This is attributed to a lower and narrower distribution of lordotic posture throughout the day in LBP patients (Figure 4). Despite the smaller lumbar flexion angles relative to the reference upright standing posture, and the resulting passive moment contributions, drawing conclusions about spinal compression forces remains premature. This is primarily due to the lack of detailed knowledge on muscle forces, especially the higher co-activation expected in the LBP population. Interestingly, despite the smaller changes in the mean lumbar flexion angle, the shape of the lumbar spine during daily activities remains basically comparable in both populations due to the lower lordosis at the initial reference upright posture in LBP patients. Pervious laboratory-model studies have reported that LBP patients experience 11% greater compression and 18% greater shear spinal loads during lifting tasks compared to asymptomatic controls, despite compromised kinematics (Marras et al., 2004). Furthermore, unstable lifting activities caused significantly, albeit slightly, larger peak compressive loads at L5-S1 (4677 N vs. 4446 N, *p* = 0.021) and L4-L5 (4567 N vs. 4366 N) in LBP patients (Heidari et al., 2022). In both studies, LBP patients exhibited increased torso muscle co-activation, resulting in greater internal loads. Previous research further suggests that motor control adaptations, such as disrupted or reduced lumbar proprioception, play a significant role in trunk kinematics and spinal loads. This can include loss of trunk control and enhanced trunk muscle co-contractions, leading to sustained mechanical loading on spinal tissues and contributing to chronic or recurrent LBP (Meier et al., 2019). It is important to note that laboratory lifting analyses are not directly transferable to daily life, as daily activities in industrialized countries predominantly involve sedentary postures.

Systematic reviews emphasize the current challenges and uncertainties associated with employing kinematic and kinetic measures to guide clinical decisions in patients suffering from LBP (Papi et al., 2018; Moissenet et al., 2021). The reviews underscored the absence of consistent findings concerning identifiable kinematic or kinetic abnormalities that can consistently distinguish individuals with LBP from those without, particularly in short-term clinical assessments. Our study supports findings by Taylor et al. (2004), who demonstrated that patient-specific dynamic analysis could lead to more accurate diagnostics and personalized treatment plans. The present and Taylors findings advocate for the use of continuous monitoring devices to capture real-time spinal movements and loads.

Similar to the kinematic measurements, the estimated bending and torsional moments have certain limitations that need to be discussed. Notably, age- and degeneration-related changes were not incorporated into the present model simulations. Disc degeneration, for instance, result in a narrowed disc with reduced nucleus pulposus and multiple defects in the annulus fibrosus, potentially leading to increased stiffness (Ghezelbash et al., 2020). Conversely, in a degenerated disc with height loss and greater axial compression, intervertebral ligaments may become lax (Adams et al., 1987; Adams et al., 2000; Adams and Roughley, 2006), thus reducing bending stiffness. Such biomechanical alterations in the intervertebral disc require attention to the level of disc degeneration before estimating segmental bending and torsional moments *in vivo*. Despite extensive research efforts, the mechanics of disc degeneration remain incompletely understood, and subject to debate, with studies reporting both decreased (Mimura et al., 1994) and increased (Kirkaldy-Willis and Farfan, 1982) flexibility *in vitro*. Consequently, disc age and degeneration were not considered in the present study. Furthermore, the moment-angle data utilized in this study (Shirazi-Adl, 2006; Heuer et al., 2007) to estimate lumbar and segmental moments were derived under specific applied loads and axial compression forces, which may differ significantly from the variable loads and motions experienced during daily activities. Additionally, the initial segmental-lumbar rotations and moments in the upright posture (El-Rich et al., 2004), which can vary between individuals, were not accounted for in this study.

## 5 Implications

The shift from static to dynamic diagnostic assessments could revolutionize the *diagnosis*, *treatment* and *management* of LBP by providing a more accurate and holistic view of spinal and back function.

1. *Diagnosis*: By revealing abnormalities and dysfunctions not observable in static positions, dynamic assessments can improve early detection and intervention strategies.
2. *Treatment*: Dynamic assessments provide clinicians with a thorough understanding of patients’ spinal mechanics over time, enabling tailored rehabilitation programs and potentially enhancing surgical outcomes.
3. *Management*: By monitoring how patients’ spines respond to daily activities, dynamic assessments facilitate feedback via wearable technology. This capability allows for adjustments in patient behaviors to mitigate pain triggers, objectively evaluates treatment efficacy, and supports preventive care strategies for sustaining spinal health and preventing LBP recurrence.

These advancements aim to achieve better patient outcomes and higher satisfaction rates. Integrating these methods into clinical practice will necessitate updating current diagnostic protocols and educating healthcare providers on the advantages and implementation of dynamic assessment technologies.

## 6 Conclusion

The current results highlight significant daily variations in lumbar spinal posture and internal bending and torsional moments compared to the static snapshot data commonly collected and used in clinical practice. Consistent with our hypotheses, both asymptomatic subjects and LBP patients exhibit a much more flexed lumbar spine with lower lordosis during daily activities compared to the upright standing posture. Furthermore, the LBP population demonstrates significantly lower lordosis (at snapshot assessment), reduced movement variations, and consequently internal moments during daily activities compared to healthy counterparts. However, due to the less lordotic posture in upright standing among LBP population, the average daily shape of both groups is found to be comparable. Additionally, sex appears to play a minor role in the recorded spinal posture.

## Data Availability

The original contributions presented in the study are included in the article/Supplementary Material, further inquiries can be directed to the corresponding author.
