# Supplementary material for: Marked differences between continuous long-term and clinical snapshot examinations: is the current standard of back pain diagnostics outdated?
Source: Front Bioeng Biotechnol. 2024 Jul 25;12:1411958. doi: 10.3389/fbioe.2024.1411958 (PMC11306014; doi:10.3389/fbioe.2024.1411958)
Supplement: Supplementary file 1 [file DataSheet1.docx]

**Additional information about Epionics SPINE system**

The Epionics SPINE system is a diagnostic tool developed by Epionics Medical GmbH (Potsdam, Germany). This system is designed to measure and analyze the kinematics and kinetics of the lumbar spine. Here's a detailed overview:

**Key Features and Functionality**

1. **Measurement Technology**:
   - The system utilizes differential strain-gauge elements to accurately measure lumbar spinal shape and rotations in both the sagittal and transverse planes.
   - It consists of two flexible sensor strips, each containing twelve 2.5-cm-long segments. These strips are inserted into hollow paravertebral plasters attached to the patient's back, 7.5 cm away from the mid-line of the spinal column on each side.
2. **Data Collection and Analysis**:
   - A tri-axial accelerometer is located at the lower end of each sensor strip, aligned with the posterior superior iliac spine, to estimate the sacrum orientation.
   - The system connects to a storage unit that collects data at a frequency of 50 Hz. This unit is relatively small (12.5 cm × 5.5 cm) and lightweight (80 g), making it convenient for continuous monitoring over extended periods.
   - The collected data is used to continuously calculate changes in the lumbar lordosis angle and other segmental angles, allowing for detailed analysis of spinal movements and postures throughout the day.
3. **Clinical Applications**:
   - The Epionics SPINE system is particularly useful for assessing the dynamic behavior of the lumbar spine in both asymptomatic individuals and patients with low back pain (LBP).
   - It helps in understanding the impact of various activities on spinal mechanics, which is crucial for diagnosing and treating spinal disorders.
   - The system's ability to provide detailed, continuous measurements offers a more comprehensive view of spinal function compared to traditional static clinical examinations.
4. **Accuracy and Reliability**:
   - The system has been validated for high accuracy and repeatability, with interclass correlation coefficients (ICC) greater than 0.98, indicating excellent test-retest reliability.

**Clinical and Research Use**

The Epionics SPINE system is employed in both clinical settings and research studies to improve the understanding and treatment of lumbar spine disorders. It is particularly valuable for:

- Monitoring the effects of different therapeutic interventions.
- Evaluating the progression of degenerative spinal conditions.
- Investigating the relationship between spinal kinematics and various pain syndromes.

By providing detailed and continuous data on spinal movements, the Epionics SPINE system supports the development of personalized treatment plans and contributes to advancing research in spinal health and disease.

**References**

Consmüller T, Rohlmann A, Weinland D, Druschel C, Duda GN, et al. (2012) Comparative evaluation of a novel measurement tool to assess lumbar spine posture and range of motion. European Spine Journal 21: 2170–2180. doi:10.1007/s00586-012-2312-1.

Consmüller T, Rohlmann A, Weinland D, Druschel C, Duda GN, et al. (2012) Velocity of lordosis angle during spinal flexion and extension. PloS one 7: e50135. doi:10.1371/journal.pone.0050135.

Taylor WR, Consmüller T, Rohlmann A (2010) A novel system for the dynamic assessment of back shape. Medical engineering & physics 32: 1080–1083. doi:10.1016/j.medengphy.2010.07.011.

| 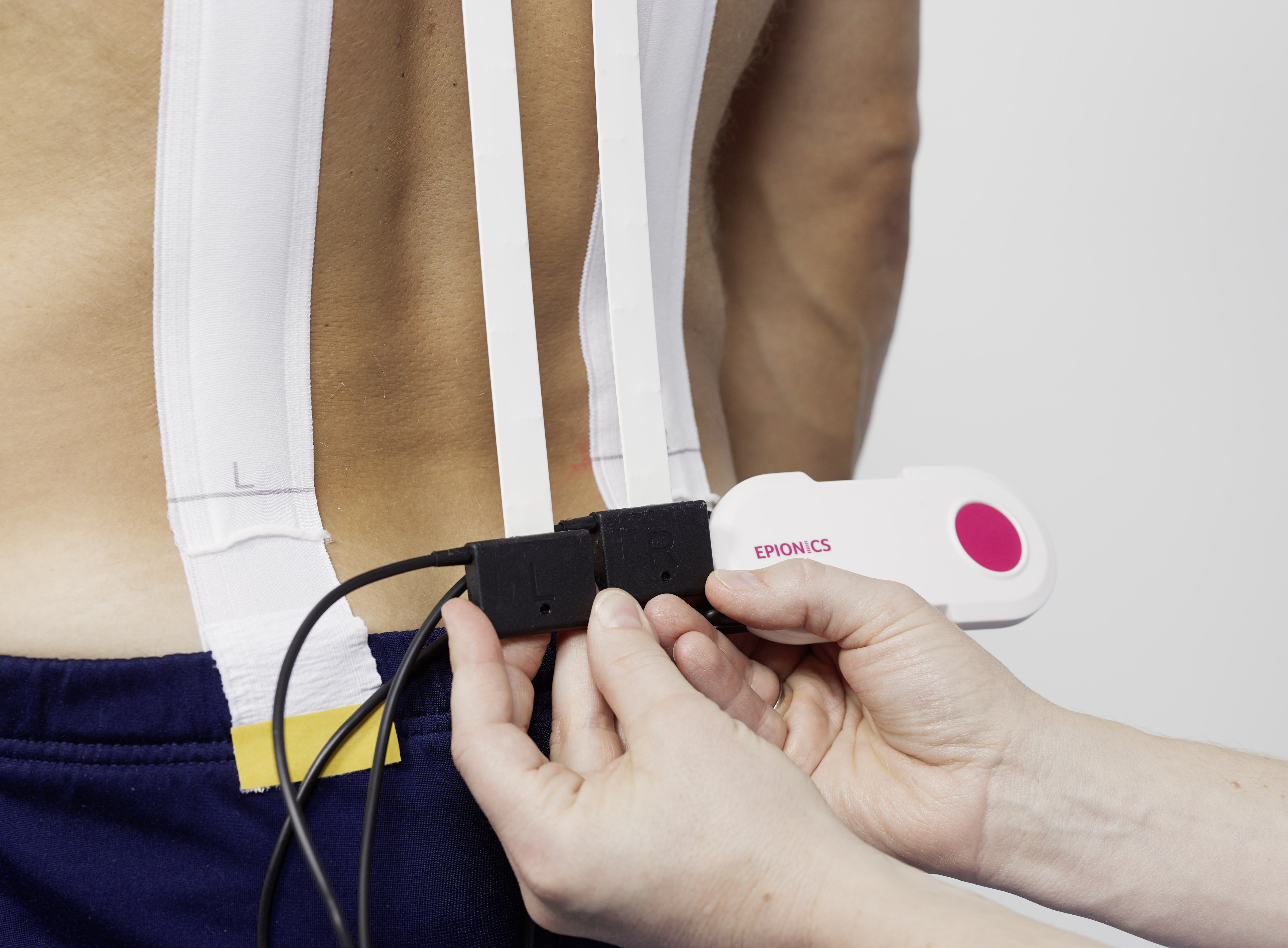 | 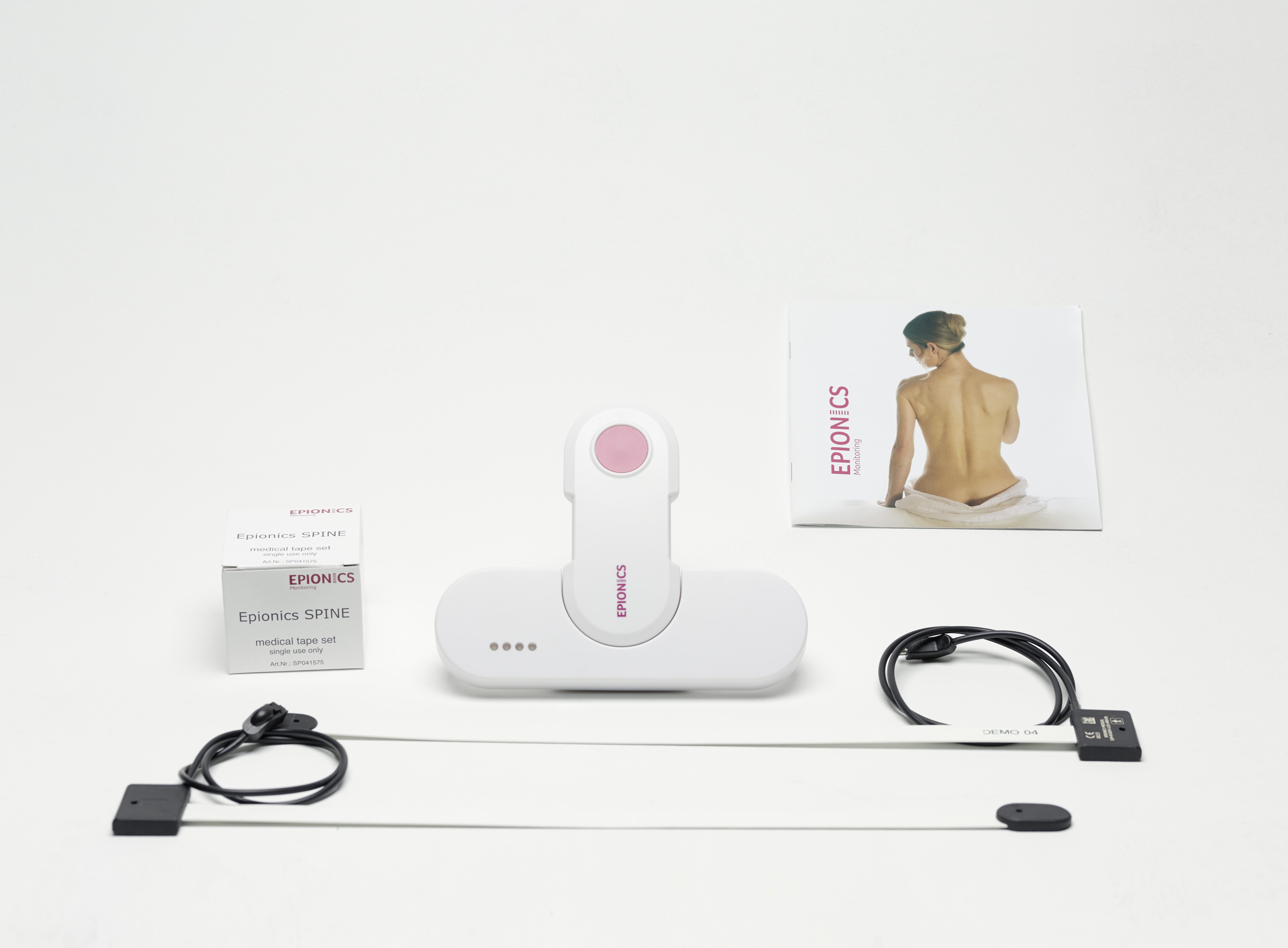 | 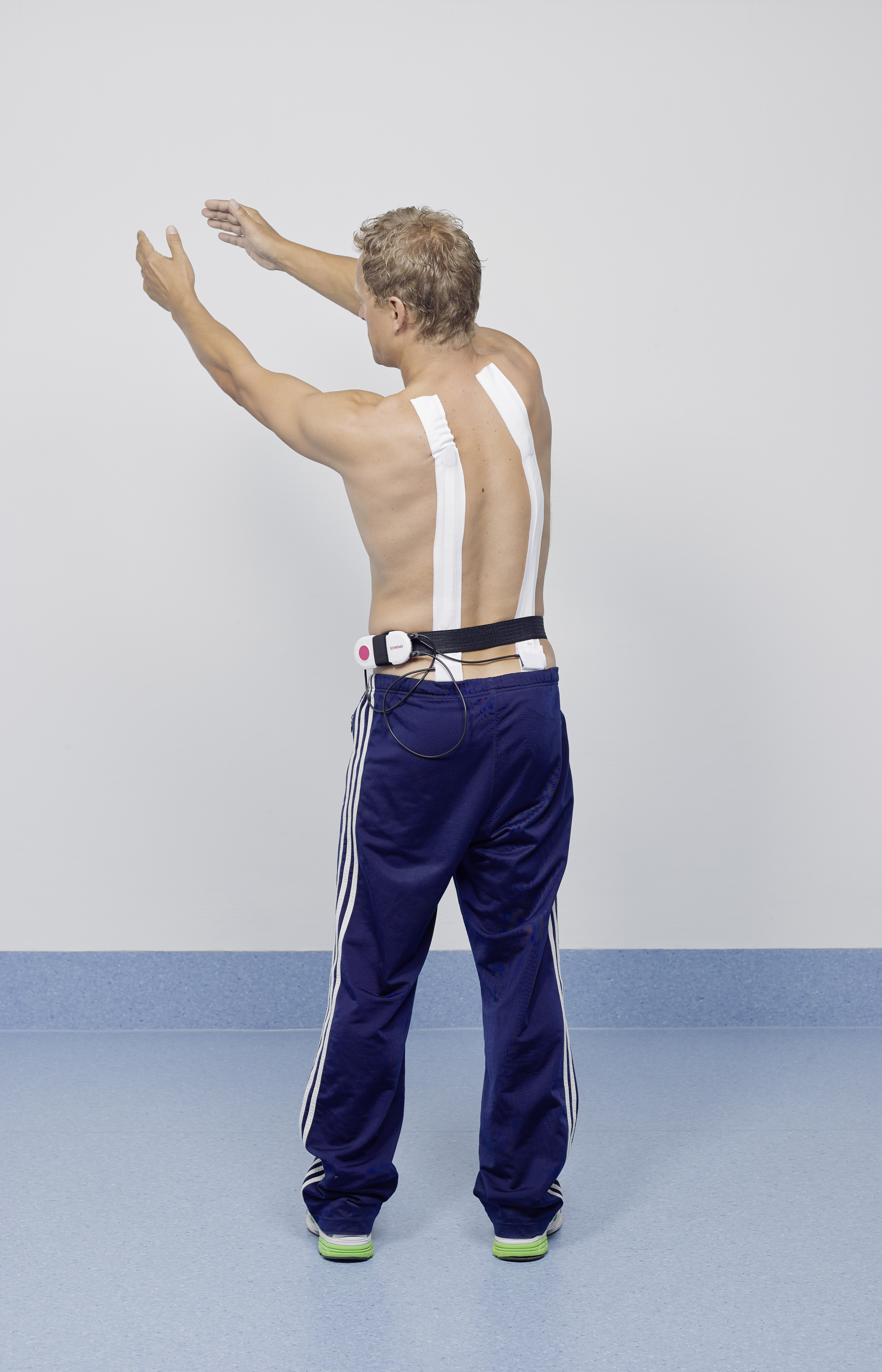 |
| --- | --- | --- |
| Suppl Figure 1: Epionics SPINE system | | |
